# A Case of Escherichia coli Endogenous Panophthalmitis and Orbital Cellulitis With Normal Workup for Primary Focus

**DOI:** 10.7759/cureus.15103

**Published:** 2021-05-18

**Authors:** Fadi F Hassanin, Sahar Elkhamary, Rawan Al Thaqib, Diego Strianese

**Affiliations:** 1 Ophthalmology, King Khaled Eye Specialist Hospital, Riyadh, SAU; 2 College of Medicine, Jeddah University, Jeddah, SAU; 3 Radiology, King Khaled Eye Specialist Hospital, Riyadh, SAU

**Keywords:** endogenous endophthalmitis, panophthalmitis, e-coli, esbl, evisceration, orbital cellulitis

## Abstract

We describe a case of a poorly controlled diabetic patient with left endogenous panophthalmitis with orbital cellulitis and positive ocular culture of *Escherichia coli *with negative systemic workup and rare clinical presentation. Was misdiagnosed and mismanaged as acute angle-closure glaucoma. Despite medical treatment with intravenous antibiotics, the patient required evisceration of the left eye as a result of the delay in diagnosis and treatment. A high index of suspicion for endogenous endophthalmitis and awareness of the proper workup and different clinical presentations is needed to avoid vision and life-threatening consequences.

## Introduction

Endophthalmitis is defined as any inflammation of the internal ocular structures it could be exogenous or endogenous. The majority of cases are exogenous such as acute postoperative endophthalmitis, traumatic or delayed onset endophthalmitis. Endogenous bacterial endophthalmitis (EBE) which is also termed metastatic endophthalmitis accounts for about 2%-6% of all cases and occurs when an organism infects ocular tissues after penetrating the blood ocular barriers during bacteremia [1-3]. Panophthalmitis results in the most extensive ocular involvement in endophthalmitis with inflammation in periocular tissues. Panophthalmitis is usually caused by very virulent organisms. The disease develops so rapidly, and the visual prognosis is very poor [4]. Organisms in EBE usually metastasize to the choroid resulting in a choroidal abscess, with delayed treatment it will progress to panophthalmitis with scleral involvement, abscess, melting, and even perforation [4].

## Case presentation

A 71-year-old female patient, with poorly controlled type 2 diabetes mellitus (DM) on oral hypoglycemic medications was referred to our hospital as a case of left eye endophthalmitis. The patient had undergone phacoemulsification one year ago and penetrating keratoplasty five years previously in the right eye. Her presenting complaint was pain and severe swelling of the left eye for five days duration with limitation of ocular motility, followed by a gradual reduction in the vision to hand motion (HM) for which she was admitted elsewhere and misdiagnosed initially as a case of acute angle-closure glaucoma (AACG) with intraocular pressure (IOP) of 48 mmHg. At that time, the patient received full antiglaucoma treatment including intravenous mannitol and Diamox, and YAG peripheral iridotomy was performed but (IOP) was not controlled (41 mmHg). Initial B-scan ultrasonography showed vitreous hemorrhage and T-sign with no evidence of endophthalmitis. However, later, computed tomography (CT) scan findings indicated orbital cellulitis and magnetic resonance imaging (MRI) showed endophthalmitis with subchoroidal abscess formation. Subsequently, intravenous vancomycin and ceftazidime were started for two days followed by oral antibiotics.

**Figure 1:**
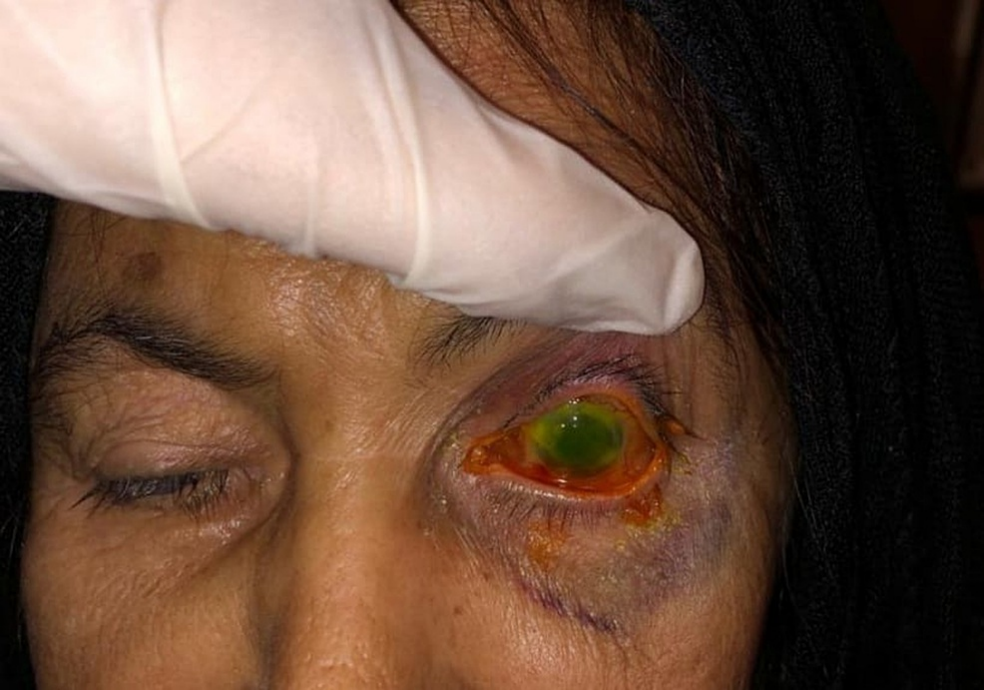
External photo of the right eye showing 360° conjunctival chemosis with hypopyon consistent with endophthalmitis. A written informed consent obtained from the patient to use the photo for publication.

On presentation to our hospital, the patient was completely blind in the left eye and complaining of tenderness and pain with mild swelling in the left eye. Review of systems was unremarkable including gastrointestinal, genitourinary, nervous, and respiratory systems. On examination, the patient was vitally stable with light perception (LP) vision in the right eye, a quiet eye with a failed graft and pupillary membrane, with no further view to the fundus. Visual acuity of the left eye was no light perception (NLP), moderate lid swelling with conjunctival chemosis, mild proptosis, and frozen globe (Figure 1).

Slit-lamp examination of the left eye showed severe conjunctival congestion, chemosis, and discharge, scleral thinning with melting was noted superiorly, opaque vascularized cornea, flat anterior chamber with iridocorneal touch, and dense cataract with no further view to the fundus. The dental exam indicated very poor oral hygiene with white oral thrush; no black discolorations were noticed. The ear and nose examinations were unremarkable.

**Figure 2:**
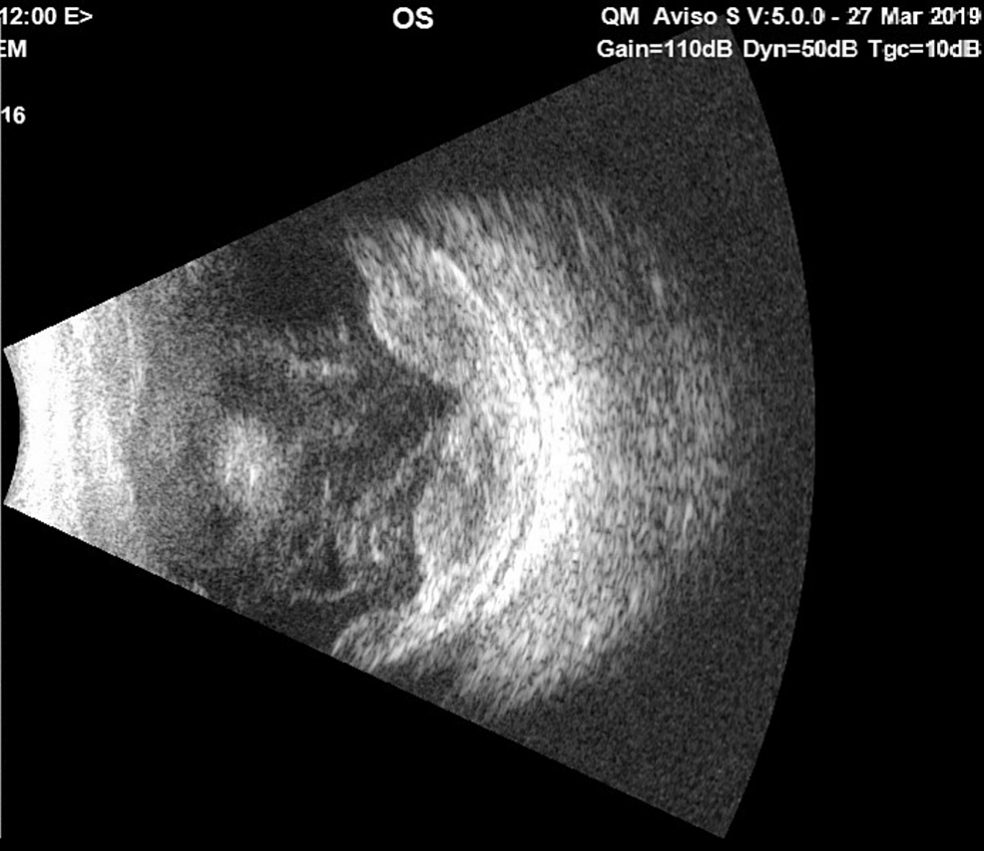
B-Scan ultrasonography of the eye at presentation showed choroidal detachment with subchoroidal opacity, vitreous strands, and scleral thickening.

The B-Scan repeated at our hospital and the left eye had moderate to dense vitreous opacities with dense vitreal strand formation, 360° peripheral bullous choroidal detachment with maximum elevation superiorly, and dense subchoroidal opacities (Figure 2).

**Figure 3:**
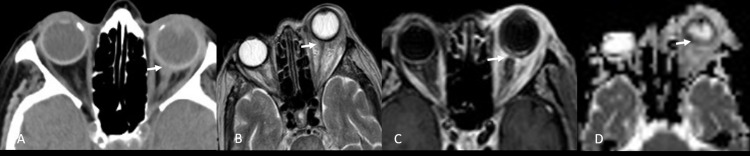
(A) Unenhanced axial computed tomography of the orbits demonstrates marked inflammatory changes involving the left globe and extending along the optic nerve. Proptosis and pre-septal edema. (B, C) Axial T2-weighted, postcontrast, fat-saturated image shows extensive enhancement of the periorbital soft tissue, detached choroid leaflet with generalized scleral thickening, irregular enhancing posterior choroid and optic nerve with enhanced chorio-retinal and scleral layers as well as intense ill-defined enhancement seen at the retro-scleral fat, along the optic nerve and ipsilateral extraocular muscles. (D) Diffusion-weighted and apparent diffusion coefficient (ADC) images showed restricted diffusion in the left-side subretinal fluid, compatible with an abscess collection "arrow", intraocular infection, and panophthalmitis.

The right eye showed no significant findings. CT and MRI images brought by the patient were reviewed at our hospital and indicated a clear picture of panophthalmitis with orbital cellulitis (Figure 3A-D).

The clinical impression was endogenous panophthalmitis with orbital cellulitis in the left blind, painful eye. The patient admitted and started on intravenous gentamycin 60 mg and, cefazoline 1 g (every eight hours), oral paracetamol 1 g (four times daily), oral acetazolamide 250 mg (twice daily), and topical moxifloxacin (every four hours), brimonidine (three-time daily), and bimatoprost (once daily at bedtime). Blood and urine samples extracted for culture and analysis. Because of the presented vision of NLP and late presentation, no globe salvage intervention performed, and the patient underwent evisceration of the left eye fortified by gentamycin wash for the inner scleral coat, without intraocular implant. Samples from all ocular layers were sent for culture, gram staining, and histopathology.

Culture and sensitivity result from cornea, sclera, and uvea indicated *Escherichia coli* sensitive to gentamycin. Blood cultures and urine cultures showed no growth. Histopathology findings were consistent with endophthalmitis.

Postoperatively the patient was vitally stable and intravenous antibiotics were continued for five additional days. The patient was referred to a general hospital for further systemic workup. Systemic workup was performed at the general hospital by the infectious disease team and no focus for the bacteremia was identified.

## Discussion

EBE most commonly affects immunocompromised patients with DM as the most common predisposing factor, other factors include intravenous drug abuse, organ transplant, cancers, and HIV/AIDS. However, it can occur in immunocompetent patients [1]. In our study, poorly controlled DM was the only risk factor identified.

Although 90% of patients with EBE have other infectious systemic conditions, 10% can occur in isolation, blood culture is positive in 54% of patients, the primary source of infection might remain unidentified in up to 40% of cases [2,4,5]. Our patient had isolated panophthalmitis with normal blood, urine, and systemic workup.

EBE can be easily misdiagnosed in 22% of patients as non-infectious acute anterior uveitis, AACG, and fungal endophthalmitis [4]. Misdiagnosis is likely due to the rare nature of this infection and the lack of history of previous surgery or trauma [4,6]. It can sometimes be overlooked in severely sick patients as conjunctivitis [4,5]. Our case was misdiagnosed and mismanaged as AACG that delayed the proper diagnosis and management for about 6 days resulting in worse clinical outcomes. The literature showed an average delay in the diagnosis of 9.5 days, which subsequently affects both visual and life outcomes [1,4]. For these reasons, clinicians should have a very high index of suspicion to ensure a timely diagnosis and refer or manage these cases.

Many organisms have been implicated in the pathogenesis of EBE. In our case, the patient was culture-positive for E. coli. Irvine et al have reported *E. coli* as the leading cause of ocular infection in patients with endogenous infections in their study [7]. Gram-negative isolates account for more than 60% of Asian cases, gram-positive isolates are more common in Europe and North America, with a predominance of Klebsiella species (spp.) and E. coli worldwide in many series [8-10]. Multidrug-resistant organisms and extended-spectrum B lactam (ESBL) producing *E. coli* and Klebsiella are reported to cause EBE [2,11].

Clinical features of EBE were very similar to exogenous endophthalmitis with pain and decreased vision as the most common ocular symptoms, loss of red reflex, and fundus view as the most common clinical signs [1,4,6]. In our case, we report one of the rarest presentations of EBE as the patient presented with a clinical picture of orbital cellulitis which was then complicated by EBE and then panophthalmitis. To our knowledge, this is the only case in the literature with this presentation.

Imaging studies with B-scan ultrasonography can be very helpful in diagnosing EBE especially if there is no view to the fundus. Further imaging by CT and MRI can confirm the diagnosis and rule out the possibility of more devastating panophthalmitis and orbital cellulitis [12,13]. Our study supports the importance of early imaging with CT and/or MRI to aid in the diagnosis, and to rely on the B-Scan only if an expert technician is available.

Blood cultures are mandatory and performing a full septic screen is indicated to identify the primary focus of the infection [4]. However, a negative workup will not rule it out as the condition may be due to only transient bacteremia or as isolated [1,2,4]. Culture of the ocular contents by vitreous or anterior chamber tap is very useful particularly in patients with negative blood cultures and those who progress despite systemic antibiotic therapy and showed a positive result in more than 60% of patients [1,2,4]. The negative systemic workup in our case could be explained by partial treatment received in another hospital or just an isolated EBE from transient bacteremia.

Patients with endogenous panophthalmitis with orbital cellulitis and NLP vision require broad-spectrum systemic antibiotics, as documented for our patient [14]. The role of intravitreal antibiotics and pars plana vitrectomy versus evisceration/ enucleation depends on the vision at presentation and patient comorbidities [14]. Evisceration or enucleation used to end-stage treatments for patients with no visual potential or rapidly progressing cases despite other therapeutic or surgical measures [14].

There are multiple reports for globe salvage intervention in case of severe panophthalmitis with a scleral abscess in NLP eyes by giving multiple intravitreal antibiotics and dexamethasone injection with very promising outcomes [14]. There are significant advantages of total resolution of the ophthalmitis, including, less psychological trauma and better quality of life if the eye is preserved [14]. Putting an implant in the primary surgery is not recommended because of the higher risk of extrusion due to the thin necrotic, melted sclera [15,16]. Due to the superior scleral melting in our patient, we preferred to proceed directly to evisceration without an implant as placing a primary implant carries a high risk of extrusion and other complications.

## Conclusions

Endogenous panophthalmitis is a rare, serious sequela of EBE and requires urgent treatment. Rarely, it can present similar to orbital cellulitis, which may later be complicated by panophthalmitis. *E. coli* is considered a rare but very virulent organism with many multidrug resistance species. Patient workup is mandatory to identify the source of the infection. A negative workup does not rule out the disease. A high index of suspension is warranted. At the early stages, intraocular injection and intravenous injection of antibiotics are useful. Evisceration is still one of the treatment modalities in patients with a late presentation, severe infection, or NLP vision. However, further studies are required to prove the efficacy of globe salvage intervention in NLP patients with panophthalmitis.
